# Discussions on Various Subjects

**Published:** 1904-08

**Authors:** 


### DISCUSSIONS ON VARIOUS SUBJECTS.

Dr. Tuller.—I, of course, have had years of experience in the use of arsenical preparations in the treatment of teeth we have to devitalize, and I came to the conclusion many years ago that some means to get along without it was very desirable. I have not used it in the past several years, except in a few instances where I have failed to get results with pressure anaesthesia, and in some of those cases—most of them, I believe—I failed with the arsenic as well, to reduce the pulps to insensibility. We have a lot of unpleasant things to deal with when we undertake to use arsenic, and among the things are aching teeth before the arsenic gets in its deadly work, and time consumed, not to mention the dangers of discoloration often a sequel to the arsenic method. In pressure anaesthesia I have met with stubborn cases and to such an extent that I have given up and fell back on arsenical treatment. I had an experience within the past sixty days. I had a patient with pulp exposures, one in a molar and one in a bicuspid. Both appeared to be favorable to the use of the instrument I so often use in pressure anaesthesia, and I applied it very thoroughly without any success in either case. I put in arsenic and sent patient away for forty-eight hours. When she returned I found pulp quite as sensitive as before. I applied the cocaine again under pressure, and after considerable time got into the pulp chamber. In both teeth I found a calcific deposit filling the chambers and extending a ways into the roots, terminating in each root in a live pulp. This deposit was not attached to the tooth at all, but filled the chamber with the exception of a thin peripheral space seemingly filled with serum or protoplasm separating it from tooth walls.

With these two cases in one mouth and some other cases where I had knowledge of secondary deposit of dentine, I am led to the belief that this deposit may be the main cause of pressure anaesthesia failures, when we are satisfied that the fault does not lie in the method used, for any pressure method used that does not absolutely confine our solution to the tissue we wish to penetrate will fail. When we know we have not failed in confining the solution and the case is stubborn we may suspect a layer of secondary dentine (which will be penetrated if we persist) or a calcified pulp which does not seem to be affected at all; anyway in any reasonable time.

To produce anaesthesia in a tooth pulp by my method I do not go into the cavity of decay at all in many cases, but make a small hole through the enamel at the most available and at the same time least conspicuous point and apply the cocaine with pressure to the tubules of healthy dentine. The solution soon penetrates the pulp, producing anaesthesia that benumbs the whole coronal portion of the tooth; then I can bore into the cavity without pain and uncover the pulp so that I can insure pressing cocaine to the apex of roots. When that is done removal follows immediately. The flow of blood can be checked with extract of the suprarenal gland, or by using it with the cocaine. It infiltrates the pulp and prevents much bleeding. Now these openings through the enamel are made at points, when possible, that will be embraced in the subsequent extension of the cavity for prevention, or for anchorage. In molars and bicuspids I can usually make this opening occlusally. Sometimes I push the gum up a little at neck of tooth and make the opening there. The selection of this point I believe often obviates the delay caused by secondary dentine. Whatever way you may get in and affect the pulp a little, you will find you can then cut in in any part of the coronal portion of the tooth with no pain. In a clinic before the Illinois State Dental Society at the last meeting, we failed for fully half an hour to get anaesthetic results, but perseverance was rewarded, and when the tooth was opened it was discovered that there was a receded pulp with the chamber walls thickened with secondary dentine.

So we have these difficulties to contend with in pressure anaesthesia, but are they more or worse than the difficulties, in one way and another, we have always had to contend with? I think not; and when we are successful there is no better way or time to get out a pulp than while yet really vital and before any putrefactive changes have taken place, or septic influences introduced. Filling immediately may be done or not as each operator deems best, but it can be done in a day or so at most, almost always, and with no occasion to introduce anything into the tooth that might discolor it we eliminate many chances of the tooth ever becoming dark.

I have diverged some from the subject in describing ways and means of pressure anaesthesia because it is comparatively new, but the way to do it is quite as important as to know that it is the thing to be done. If you agree with the essayist that the immediate removal by some cataphoric process is the thing to do, you want to know how to do it, so I may be excused possibly for the divergence. When the case in hand will permit of the use of a soft rubber plug behind the pellet of cotton saturated with the cocaine preparation —no leaking out—nothing better is needed, but there are so many cases where that procedure does no good at all, because solution leaks away.

Dr. C. S. Stockton, Newark, N. J.—We, as a profession, are not alone in the difficulties which are surrounding us. The lawyers in a measure suffer from similar troubles. Our loved State of New Jersey occupies an anomalous position, being the from another State cannot practice law only State in the Union in which a lawyer without an examination.

A lawyer from Pennsylvania who has been in practice before the higher courts for three years can secure from a judge of that court a letter which he can take with him to the State of New York and have some friend move, on that letter, that he be admitted to practice before the courts of that State, and that is universally done. I mention those two States merely as an example of all others. But in New Jersey a lawyer must pass an examination before being allowed to practice. Our medical friends are in no better condition, for they cannot go into other States under any reciprocal arrangement; they must pass an examination. A very prominent physician within the last few days said to me, "We are looking to you (that is to the dentists) to solve the problem of reciprocity for us." So you see our action is looked forward to with interest not only by dentists but by others.

I brought forward this morning in a measure a scheme which I thought might be a successful solution of this vexed problem, and some of you heard it, but it will not hurt you if I repeat it tonight, and those who were not here may possibly be interested. Let us take the familiar case of Dr. Osmun who is known to us all favorably and well. He has gone to California; suppose that he desires to practice dentistry there. Under my plan I would say to him, "You shall come before the Board of Examiners of the State of New Jersey and obtain from that Board a certificate, not taking the old one you got years ago, but taking one now, stating the fact that you are a reputable dentist, that you have been in practice for twenty-five years or more and that you are a good citizen." Dr. Osmun would then take that certificate to California, deposit it with the Examining Board of that State and ask for the privilege of practicing there. My point is that those gentlemen of California, knowing the character of the Examining Board of New Jersey and the high standard they maintain, and the kind of men they turn out, and knowing that the certificate given by them to Dr. Osmun is something like a letter of introduction, will look at it in that light and say, "The New Jersey Board has given to Dr. Osmun a certificate of his character now, not one of years ago, but he is all they say at this time, and, as a matter of courtesy, he has the right because he has gone through all the preliminary and necessary examinations and qualifications, to practice dentistry, and he ought to have the right to go and practice, if he knows how, wherever he pleases over this broad land."

That seems to me to be a movement which can be carried out, I do not see why it cannot be. It seems to me a much more feasible plan than that of asking the legislatures to pass a uniform amendment, which I doubt very much the practicability of doing. It seems to me there is nothing in the way of accomplishing my plan and the New Jersey Board would be glad, if any one came to it with such a qualification as I have mentioned, to say to him, "Come into our borders, practice dentistry and we will extend to you the right hand of fellowship."

Dr. Emory A. Bryant, Washington, D. C.—This question came up nine years ago, the first time I had the pleasure of meeting with the New Jersey Society. I was then talking of just what my friend, Dr. Stockton, has been talking tonight, reciprocity, and I can say that I agree thoroughly with all of the gentlemen who have preceded me.

I have had a little experience in my life in this very line and the nine years that have passed since my first appearance before this society have not changed my ideas very much. Dr. Kirk's idea is very fine and would be practical if it did not hit the commercial side and furthermore if it did not legislate out of office some gentlemen who hold offices. Dr. Ottolengui's idea finds trouble in the same line. He said if the National Board of Dental Examiners would do so and so, they could get the law. The National Board of Dental Examiners will never do that. they might legislate themselves out of office if they did.

If you want a movement of this kind to succeed you must get the men of the different States interested in the subject, outside of the colleges and the Boards of Examiners. If you want any action taken you must get new men at the helm. They have been talking the same thing for the last ten years, ever since the National Board of Examiners was organized, and they have not done a thing. We have had reciprocity in New Jersey and in New York and that has broken up, practically. You have had reciprocity with other States and that never amounted to anything. Why? Because you have waited for some Board of Examiners to take the matter up. I have never seen a man in my lifetime, since I have been in dentistry, who had an office and wanted to get rid of it. Office holders hang on like grim death.

Dr. Osmun has gone to California, if I mistake not the Board of Examiners of the State of California would require him to pass an examination today after perhaps twenty-five or thirty years' practice, just as they would require it of a young man just coming out of school. The certificate from the State Board of New Jersey would have absolutely no effect except, as Dr. Stockton says, as a matter of courtesy. If they would accept these things as a matter of courtesy it would be all right, but they will not, and the same commercialism comes in again, "We don't want you to come into competition with us, if you are a good man in the State of New Jersey, we don't want you in our State, you will take something away from us." I was told in the city of New York by a man who stands as high in the profession today as any man in that city, that New York did not like new dentists, and that if a certain dentist came there he would not receive any aid whatever. Why one additional man in the great city of New York would be like a grain of sand thrown in the ocean, there would be a little ripple and that would be the end of it.

If you have any idea of getting reciprocity throughout the country you must take hold of new men who are willing to work and let them get out and hustle; let them appear before the State legislatures and you may get what you want but you will never get it from the colleges, through the State Boards of Examiners or through the National Association of Dental Examiners.

Dr. A. W. Harlan, Chicago, Ill.—I have been listening to the discussion that has taken place this evening and it seems to me that under the constitution that we live under and the rules that govern our States, the only feasible method is to have amendments to our present dental laws. If we have uniform laws throughout the country so that the standard would be practically the same, we might have interchangeable certificates.

Some of you know that in Switzerland, which is divided into a number of Cantons, a man is not permitted to practice throughout the entire country. Certificates are issued for the canton of Geneva, or Lucerne, or whatever the name of it may be, while in all foreign countries the requirements for practice are becoming so that examinations have to be taken in the language of the country, and furthermore men cannot take examinations unless they have the qualifications of an A. B. or B. S. or Ph.B. or Ph.D. degree; so that very shortly all Americans who desire to practice in any foreign country will have to attend their schools and pass their preliminary requirements for entrance into their schools. In the United States, while it would be very desirable for a gentleman in New Jersey to practice in New York, if the standard is not the same for examination or with reference to the recognizing of diplomas I think the State police regulations will still be maintained. Courtesy might be abused. In some States, Boards of Examiners are appointed without reference to their fitness for holding such positions. I was a member of the first Board of Examiners in the State of Illinois, appointed in 1881, and I served for five years and later was again a member and we had applications for registration of certificates from States, many of which were new, and as we found out were not based on proper requirements at all. There were certificates which were bought and some which were given on such slight examinations that it would have been an injustice to the people of the State in which I resided for our Board to extend any courtesy to the holders of such licenses.

Dr. James R. Piper.—Case I: Some few years ago I had occasion to restore a lost lateral, on the left side, and in looking about to see how this restoration was to be made I was at a loss to find a particular system that would apply to this case. Gold bands are unsightly, and backings cemented to the palatal surface of the teeth on their side of the space, has its objections. I decided upon a bar. Of course in inserting a bar with a tooth attached, it is somewhat difficult to make the attachment and make the tooth secure, and at the same time permanent. So it occurred to me that Dr. Shaw had worked out a little appliance in the form of a bar bridge which is made from a common plate tooth, with vertical pins. The artificial tooth is backed up in such a way as to make a slot running horizontally across it. In this slot is carefully fitted a bar of iridio-platinum of the right width, and a hole is drilled through the backing and bar. In that hole threads are cut and a screw made to fit. Then the bar is taken off from the bridge and the cavities (there happened to be cavities in these teeth) so shaped as to permit of securely fastening the ends of this bar; the tooth is taken off from the bar and the bar cemented in place, the cement being cut out as the work progressed for the gold filling, which was the permanent retention. After the bar had been securely packed about with gold, the slot in the backing of the tooth was filled with cement, the tooth set on the bar and the screw adjusted. In this particular case this seemed the best method for restoring the lost tooth.

Case II: The second case which I will report was a molar crown in the mouth of a lady whose teeth were somewhat conspicuous. The idea was conceived of making a wide band, just as you would for a gold crown. In the band is burnished a piece of very thin platinum (such as you would use for a matrix), allowing the platinum to come over the periphery of the gold band, thus making a little shelf to hold the porcelain from being forced into the gold band. The platinum band is removed from the gold band and porcelain cusps, made from high fusing body, baked into the platinum band, scoring the body on the under side, for retention. I have inserted them without scoring the porcelain. Then after the band is set the porcelain cusps are carried to place in cement and the teeth brought together and firmly held for ten or fifteen minutes. That worked very satisfactorily.

Case III: When Jenkins's porcelain bodies came out I had occasion to make a temporary cap for a bicuspid. I wanted to do it quickly and as gold was unsightly, I thought the appearance could be relieved by baking the cusps of Jenkins's porcelain body directly into the 22 karat band. While a very strong body for porcelain inlays, I had doubts about this being strong enough for cusps. This method was used, however, and the cusps have withstood the stress of mastication.

The same method was used on molars and bicuspids, i. e., the Jenkins body baked directly into the 22 karat band. Whether I shall be sorry for doing this I do not know. They have been in use two or three years.

Dr. R. H. Hofheinz, Rochester, N. Y. —The question of education will always remain a fruitful text for unlimited discussion for any gathering of this kind.

I have been asked to discuss the paper of our worthy President, but after looking over the epitome he sent I could find little to discuss. My views coincide so much with Dr. Patterson's that I can only reiterate his opinions and recommendations in my own language.

The Doctor divides his paper into two great halves: The deficiencies which arise (1) from a lack of education before entering college; (2) from faulty instructions after having entered college.

To accept students of inferior preliminary education is the greatest fault I can find with all our dental educational systems. This question alone should suffice for a good long essay. No additional years of college or university teaching can compensate for the defectiveness of early intellectual training.

The Germans demand nine years of gymnasium before they consider the mind ripe for university lectures. The introduction of a four years' university course can never compensate for early deficiencies. The mind must have proper shaping in youth to form an intellectual storage battery for our university teachings, and I am almost inclined to think it more important for dental students than for any other. Most of the scientific studies require a cerebral effort only; whereas, in our case, the manual, the technical development consumes much of our college time. It is the early part of life which is a period of plasticity, a period of adjustment—of a physical adjustment—and then an adjustment on a larger and broader scale.

N. M. Butler says, "There are in the United States no obstacles interposed between the college and the university. We make it very easy to pass from the one to the other; the custom is to accept any college degree for just what it means. We make it equally easy to pass from one grade, or class, to another, and from elementary school to secondary school. The barrier between secondary school and college is the only one that we insist upon retaining."

Gentlemen, it is, however, not only the quantity of preliminary knowledge which proves a telling factor in our dental education; it is the quality which our president has well emphasized.

Three years ago I chose this question for a paper before the National Dental Society. My opinion on this subject remains unchanged and I take the liberty to quote from aforesaid paper:

Manual Training: "I make the broad statement that a student who under favorable conditions has not developed some manual skill at the age of eighteen—the time for college entrance—does not possess the physical requisites of a good dental operator. In my estimation we should be in position to judge the physical the same as the mental equipment of the dental student previous to college entrance. The student who is not naturally endowed with a reasonable amount of manual ability will never become the dental operator so much needed."

Manual dexterity is but the evidence of a certain kind of mental power, and it should go hand in hand in its early development with that of the mind. This education belongs to the secondary schools, and if this quality is insisted upon as a preliminary requisite, the possibility of unfitness during college life, of which Dr. Patterson speaks, is reduced to a minimum.

Professional Ethics: Much stress is laid in Dr. Patterson's paper upon the fact that professional ideas are not sufficiently taught in colleges; that the teachers are lacking in the proper spirit of directing the students toward professional honor and ethics. I am of the opinion that you may teach ethics in dental colleges to a large number of students until

Doom's day without accomplishing the desired result.

Why? Professional instinct, reverence for scientific achievements, the proper feeling for ethical culture, belong largely to early home training and proper, elevating environments outside of a college. The young man, endowed by Nature with a spirit of commercialism, the predominating characteristic of our time, can never grasp the meaning of professional refinement at the age of twenty, no more than the man who enjoyed "rag time" music only, up to that time, can ever really appreciate the celestial meaning of a Beethoven adagio. The wrong in this direction is usually done, and permanently done, before the student enters a dental college. This, however, does not deny the necessity of ethical teaching in our colleges, and I should consider myself very inefficient in performing my duty, did I not constantly endeavor to lead the student's mind to a proper and correct understanding of professional honor.

There is no better mode of showing proper professionalism to the students than the conduct of your own practice. This is the best object lesson you can give students at that time of life. Nothing will impress the students with a higher regard for professional loftiness than the success which a teacher has to show through the application of these very principles.

All teachings of ethics in the lecture room smack of absurdity and lack sincerity, if not properly sustained by the teacher's own methods in practice. The man who cannot show through his own conduct in life the very pathway to ethical professional success, has no moral right to pose as a teacher.

Demonstrators: The other great fault Dr. Patterson finds in college teaching is the inefficient number, and, often, poor quality of demonstrators. This is certainly a most vital question. A large number of our dental colleges are without endowments, and therefore exclusively dependent upon the income from the students, a condition which necessitates economy and very often in the wrong place.

I would rather have one competent demonstrator in my infirmary than half a dozen incompetent ones. I have often endeavored to settle the relative necessity of one demonstrator to a certain amount of students, but I found it impossible, owing to the difference in the quality of students which each class presents. The consensus of opinion of our demonstrators and myself is, one demonstrator to ten students. I have repeatedly seen one student able to keep all the demonstrators busy which our university could furnish.

There is but one ideal way of teaching and that is to study the individuality of your student and teach according to its demands. If I were rich enough I should create a fund for that purpose and prove to you results such as Froebel has given to the world in his kindergarten system. I never have and never will permit any difference of infirmary practice from my method of didactic teaching. Dentistry is not a very broad profession, but it certainly permits numerous ways of execution. It is utterly impossible, without confusing the minds of beginners, to teach definitely more than one system, and that should be one based on successful and progressive methods. Other operative methods should be alluded to and teaching should thus be cosmopolitanized, but neither the mind nor the hand of a student should be allowed to wander in all directions, without a positive method, which must prove the foundation for all possible future ramifications.

If what Dr. Darby said is true, that "We made better dentists then than now," it is our duty to find the cause of such retrogression. The proper way to reach that end is by such frank presentations of our faulty methods as Dr. Patterson has given us, and by intelligent criticism of such faults.

No profession has made such wonderful progress in a short time as ours. A time for proper assimilation is needed, after which we will replace that statement of Dr. Darby's by one which will tell the world that we are making better dentists now than ever.

Dr. John I. Hart, New York City.—No system is so perfect but that it is susceptible of improvement. The fact that this body of busy men congregate here for no other purpose than the advancement and elevation of dental education is an encouragement in itself, and the most pessimistic must admit that if faults exist they have only to be recognized and their causes defined to lead to their correction.

The main difficulty lies in agreeing as to these causes.

In the last few years we have departed so rapidly and radically from early methods of dental education that practically a student of today is trained in a manner along fundamentally different lines from those which formerly existed. That this is not entirely satisfactory those who agree with the address just read must admit. Formerly, prospective dentists were received as students in an office for a certain period of time prior to entering college. This method possessed two advantages; it enabled the experienced practitioner to observe manual weaknesses in the student and to tell him and those interested in him of the advisability of his choosing another calling. It also gave the student more and greater individuality than is possible today. In this way the weeding process commenced before the student was committed to dentistry as his permanent life work.

Need of Manual Training: The number of students precludes the possibility of continuing on these lines and present methods of education have supplanted the older ones, but it throws a responsibility on dental educators which they must not and cannot shirk. Your essayist says truly that the first fault is in accepting students of inferior preliminary requirements. This proposition must be regarded in its broadest sense; we are hide bound as to the prospective student's minimum literary education, but nothing is said as to his or her manual dexterity. So long as the standard of admission into the educational department of a profession which is mechanical as well as theoretical is purely literary, so long will we have a large proportion of disappointments and failures. I would rather teach a student of ordinary literary ability, but trained manually, than a Master of Arts with no manual training. With as much reason should a professor of chemistry be expected to teach a student arithmetic so that he should be able to work out a chemical equation, as that the departments of operative and prosthetic dentistry should be required to find out, after a student has been duly entered, whether he has the manual ability to perform his required work. No extension of the number or length of courses will stand in lieu of entrance requirements.

The records of our school will show that we have in several instances advised students against continuing to study dentistry, and in some instances absolutely refused to permit them to continue with us, but how much loss and chagrin could have been saved them, if, instead of finding this out after a waste of time and money they had been stopped at the threshold. I suggest that we require evidence of manual as well as mental training before accepting dental students.

If we can prepare individuals more thoroughly, we will have less ground for fear that they will go wrong ethically. A student who has spent three years acquiring a dental diploma and is then unsuccessful in practice building, has not the moral courage to enter another calling, but, unfortunately, is attracted by methods which he may abhor.

